# Eradication of measles: remaining challenges

**DOI:** 10.1007/s00430-016-0451-4

**Published:** 2016-03-02

**Authors:** Heidemarie Holzmann, Hartmut Hengel, Matthias Tenbusch, H. W. Doerr

**Affiliations:** Department of Virology, Medical University of Vienna, Vienna, Austria; Institute of Virology, University Medical Center, Albert-Ludwigs-University Freiburg, Freiburg, Germany; Department of Molecular and Medical Virology, Ruhr-University Bochum, Bochum, Germany; Institute for Medical Virology, Goethe-University Hospital Frankfurt, Frankfurt/M., Germany

**Keywords:** Measles, Elimination, Eradication, Vaccination, Herd immunity, Surveillance

## Abstract

Measles virus (MeV) is an aerosol-borne and one of the most contagious pathogenic viruses known. Almost every MeV infection becomes clinically manifest and can lead to serious and even fatal complications, especially under conditions of malnutrition in developing countries, where still 115,000 to 160,000 patients die from measles every year. There is no specific antiviral treatment. In addition, MeV infections cause long-lasting memory B and T cell impairment, predisposing people susceptible to opportunistic infections for years. A rare, but fatal long-term consequence of measles is subacute sclerosing panencephalitis. Fifteen years ago (2001), WHO has launched a programme to eliminate measles by a worldwide vaccination strategy. This is promising, because MeV is a human-specific morbillivirus (i.e. without relevant animal reservoir), safe and potent vaccine viruses are sufficiently produced since decades for common application, and millions of vaccine doses have been used globally without any indications of safety and efficacy issues. Though the prevalence of wild-type MeV infection has decreased by >90 % in Europe, measles is still not eliminated and has even re-emerged with recurrent outbreaks in developed countries, in which effective vaccination programmes had been installed for decades. Here, we discuss the crucial factors for a worldwide elimination of MeV: (1) efficacy of current vaccines, (2) the extremely high contagiosity of MeV demanding a >95 % vaccination rate based on two doses to avoid primary vaccine failure as well as the installation of catch-up vaccination programmes to fill immunity gaps and to achieve herd immunity, (3) the implications of sporadic cases of secondary vaccine failure, (4) organisation, acceptance and drawbacks of modern vaccination campaigns, (5) waning public attention to measles, but increasing concerns from vaccine-associated adverse reactions in societies with high socio-economic standards and (6) clinical, epidemiological and virological surveillance by the use of modern laboratory diagnostics and reporting systems. By consequent implementation of carefully designed epidemiologic and prophylactic measures, it should be possible to eradicate MeV globally out of mankind, as the closely related morbillivirus of rinderpest could be successfully eliminated out of the cattle on a global scale.

## Basics

Measles is a highly contagious infectious disease caused by a RNA virus of the paramyxoviridae family in which it belongs to the genus morbillivirus. Other species of this genus are the canine distemper virus of dogs and the rinderpest virus of cattle. These morbilliviruses are strictly host-specific, i.e. there is no virus transmission from animals to humans or vice versa in terms of epidemiologic relevance [1]. The measles virus (MeV) is a spheric particle with a diameter of approximately 200 nm. A layer of matrix proteins (M) beneath a cell membrane-derived lipid envelope covers a core complex consisting of non-segmented, negative-sensed RNA genome packaged in nucleoprotein (N), large polymerase protein (L) and polymerase-associated protein (P). The glycoproteins H and F form spikes on the envelope. H mediates virus attachment to the receptors of the target cell (in vitro haemagglutination), and F mediates the fusion of the virus envelope with the cell membrane, thus releasing the core complex into the cell. Through fusion of neighboured cells (to multinuclear giant cells), the virus infection can be directly transmitted. N is abundantly produced in infected cells. N and the other MeV proteins are presented to specific cytotoxic T lymphocytes via the MHC I system, while antibodies to H and F neutralise infectivity of virions. By sequencing 450 nucleotides of the N gene (in the C terminal part), 24 genotypes compiled in eight clades (A–H) have been identified. However, cross-neutralisations with strain-specific antisera revealed only one serotype [2].

MeV is air-borne transmitted by nasopharyngeal droplets. The virus is highly lymphotropic, infecting dendritic cells, alveolar macrophages and subsets of B and T cells in the lymphoid tissue of the lower respiratory tract before it infiltrates the epithelium of the upper respiratory tract. MeV also spreads to the conjunctiva, skin and other organs, where it replicates primarily in endothelial cells, epithelial cells and macrophages [1, 2]. About eight (to 12) days post-infection, non-specific prodromi with cough, coryza and conjunctivitis develop and last for 2–4 days. At the end of this prodromal phase, the pathognomonic Koplik spots appear at the buccal mucosa. About 12 days post-infection, an erythematous maculo-papular rash erupts and spreads usually from head over trunk to the extremities. Pyrexia is common and lasts approximately 1 week. Nearly everybody who is infected falls ill. A special feature of measles is a long-lasting generalised immunosuppression due to the loss of immune memory B and T cells resulting in an increased susceptibility to other infections even several years after recovery [3, 4]. As a consequence, in many patients complications are observed, especially under the settings of malnutrition in developing countries. The majority of them are caused by bacterial superinfections, e.g. otitis media (7–9 %), pneumonia (1–6 % in industrialised countries, however, the most common cause of death in developing countries) or diarrhoea (8 %) [2, 5]. Post-infectious encephalitis occurs in approximately 0.1 % of the patients with resulting in fatal outcome in approximatively 20 % of those or leading to permanent sequelae in the majority of survivors [2]. In patients suffering from a pre-existing immunodeficiency, an “atypical” (i.e. MeV-induced) giant cell pneumonia can occur as a life-threatening event. In those patients, lethal acute progressive encephalitis is also frequently observed. The reason for this severe course of the disease is that MeV-infected cells must be eliminated by T cell immunity, which is not fully working in such infants yet or may be impaired by other diseases. If the T cell system is deficient, the patient is likely to fall ill with life-threatening “white” measles. The exanthematic skin inflammations are lacking, but the virus dissemination in the body is enhanced [2]. Moreover, a sub-acute sclerosing panencephalitis (SSPE) may develop 1–10 years after apparent recovery from measles, especially after infection in the first year of age (because of a still immature T cell system). SSPE is associated with MeV strains escaping specific (humoral) immune responses persisting in neurons and glia cells of the brain. Mutations of M, F and H proteins have been considered relevant [6, 7]. The prognosis of SSPE is lethal. The frequency has been re-evaluated in recent years and is much higher than assumed before with a reported rate of approximately 1:5000 [1]. In Germany, the risk of children contracting measles infection below 5 years of age has been calculated to be as high as 1:1700–1:3300, which is in the same order of magnitude as the risk of a fatal acute measles infection [8]. However, the big majority of measles patients recover 1–2 weeks after the onset of exanthema, but remain in the following years susceptible to secondary infections due to the long-lasting memory B and T cell impairment.

## Vaccination

Because of its high infectivity and spreading, a single person with measles infects an average of 12 to 18 people in a fully susceptible population. This is a reproductive rate (R_0_) that is much higher in comparison with Ebola with 1.5–2.5 or Influenza with 1.4–4 [5, 9]. Measles presented as a typical childhood disease before the start of infant vaccination programmes. The high morbidity had made measles a big burden to the public health. Moreover, because of relatively high rates of complications (20–30 %) and fatal outcomes with estimated 7–8 million children that died annually due to MeV infection in the pre-vaccination era, measles was commonly feared, although the majority of children recovered ad integrum. Thus, there was a big need for vaccination [1].

Vaccines were being developed soon after MeV had been isolated and propagated in human or monkey cell cultures, and available since 1963 [2, 5]. The first vaccine was an inactivated MeV strain. It did not provide a good and long-lived protection, since some of the relevant parts of the F spike on the virus envelope were slightly denatured in the inactivation process. This is why the neutralising antibodies raised by this dead vaccine did not reliably block wild-type MeV superinfection, and only provided a partial immunity (against the H spike). On the contrary, vaccine-induced antibodies sometimes enhanced the subsequent wild-type virus infection leading to a long-lasting pneumonia and death of the vaccinee (atypical measles infection) [5]. Consequently, this vaccine was replaced by attenuated live MeV of genotype A, mainly derivatives of the 1954 isolated Edmonston strain: “Further attenuated measles vaccines,” “Schwarz,” “Moraten,” etc. They had been developed by serial passages at different temperatures in human kidney, human amnion and embryonic chicken cell cultures [2, 5]. These vaccines, most often combined with mumps and rubella vaccines (MMR), were widely applied since decades and with hundreds of millions of doses administered have been proven to be well tolerated, very safe and confer long-lasting protection [9, 10]. Adverse reactions are generally mild (e.g. fever occurs in 5–15 %, rash in 3–5 % of vaccinees) [5]. Less common adverse reactions with a frequency of 1 in 1000–1 in 10,000 vaccinated individuals are lymphadenopathy, swelling of parotid gland, diarrhoea, vomiting or febrile convulsions. Rare adverse events (<1 in 10,000 vaccinated individuals) are urticaria, transient thrombocytopenia and deafness, meningitis/encephalitis in up to 1 per 100,000 or anaphylactic reactions in 1.5 per 1,000,000 vaccinated individuals. In the vast majority of countries, vaccination has been introduced in routine childhood immunization programmes with the administration of two doses. The first dose (MCV1) should be administered, as soon as protecting maternal antibodies have vanished, usually at the age of 9–12 months [11]. The second vaccination (MCV2) can be administered after a minimum interval of 4 weeks and is often recommended to be given at the age of 15–18 months or, in countries with low measles transmission and high MCV1 coverage, even later [11]. During an epidemic with an increased risk of exposure, start of MMR vaccination can be recommended for infants as young as 6–9 months of age [5, 12]. However, because immunogenicity and effectiveness are lower than for doses administered at a later age, this early vaccination should be counted as “zero dose” (recorded as MCV0) and the child should subsequently receive both routine doses at the recommended ages according to the national schedule [12]. Several studies have shown that about 95 % of vaccines produce sufficient antibodies to be protected against measles after the first vaccination. The protection rate increases up to 99 % after the second vaccination [7, 13–18].

## Global eradication of measles

From the beginning, common infant vaccination has dramatically reduced measles in all countries of the world, where programmes have been implemented consequently. For example, in the pre-vaccination era in the US measles outbreaks occurred every year with a median incidence rate of 317.1 cases per 100,000 population. After vaccine licensure (1963) and the start of vaccination programmes in 1970s, the incidence rates dropped >95 %. It has been calculated that since the introduction of the vaccine about 35 million measles cases has been prevented [19]. Because of the impact of measles on global health, the lack of an animal reservoir and the availability of live-attenuated measles vaccines providing long-term immunity after administration of two doses [5, 20], the WHO (in collaboration with UNICEF and the Measles Initiative) launched a global immunization campaign against measles aiming for a 90 % reduction in measles-related mortality by 2010 [21]. In the American WHO region, measles was eliminated by 2002 by reaching a high coverage of two doses of measles vaccine due to the full implementation of a vaccination strategy including routine and supplementary immunization activities. This was proof of principle that measles elimination is feasible and practical. Because of the success of measles-associated mortality reduction and elimination efforts, five of the six WHO regions including the European adopted regional measles elimination targets initially by 2010 and revised to 2015 [22]. This deadline could not be hold for several reasons [23] and was expanded until 2020 [24]. To reach this aim and to stop endemic MeV transmission, a seamless vaccine uptake of at least 95 % with two doses of MMR vaccine is considered to be necessary in all countries. Even in countries which reached the elimination goal, local gaps in vaccine coverage can result in outbreaks due to imported MeVs [25].

Up to date, the worldwide campaign was very promising [25, 26], leading to a 78 % decline in estimated annual deaths globally from 562,400 in the year 2000 to 114,900 in 2014. While the first dose of measles vaccine coverage increased globally from 72 to 85 % between 2000 and 2010, it has remained unchanged the past four years [25]. In Europe, the number of measles cases dropped by 98 % from 341,289 in 1993 to 7073 in 2007. However, since 2010, MeV activity rebounded with approximately 37,000 cases in 2014. In 2015, the sought elimination year in Europe, large outbreaks occurred again, and also Germany and Austria with more than 2500 and 300 cases, respectively, were affected. As a consequence of the increased MeV activity in certain countries, especially in China, the Philippines and Viet Nam, nations that have eliminated measles are confronted the last years with an increased number of importations, which led, for example, in the USA to more than 600 measles cases in the year 2014 and recently to the first measles death since 12 years. Thus, more efforts are urgently needed, which is discussed below, especially from the perspective of the European German-speaking countries.

## Challenges

In recent years, measles has had a comeback in populations in which effective vaccination programmes had been installed for decades, like in USA, Australia, England, Germany and other European countries [9, 10] This has raised some key questions:

1. Is the vaccine still efficient?
  Does primary and secondary vaccine failure matter?

The live measles vaccine strain, which is commonly applied, has been isolated from a patient and propagated in embryonic chicken cell cultures as long as random mutations have attenuated its pathogenicity. Modern molecular biologic analysis has typed most vaccine viruses as members of the extinct genotype A, derived from the MeV strain Edmonston [27]. In contrast to the highly contagious wild-type virus, the vaccine virus is not infectious to immune-competent individuals, and person-to-person transmission of vaccine virus has never been documented [5, 11]. To our current knowledge, every MeV strain starts the infection process by interaction of envelope glycoprotein H to virus-specific cell receptors, i.e. CD150, which is a signalling lymphocytic activation molecule (SLAM), and CD46, which is an inhibitory complement receptor [1]. Obviously, vaccine MeV has got a crucial mutation in the H gene which reduces its interaction with CD46. More important for the attenuation is the enhanced interferon induction of the vaccine virus in comparison with the wild type [28]. In addition, further mutations in MeV genome may contribute to attenuation. In particular, the L gene might be the target of in vitro artificial virus attenuation. Despite the fact that other MeV genotypes can replace indigenous genotypes [29], there is no evidence for immune selection of such viruses in homogenously immunised populations. Forming conserved epitopes across genotypes MeV is antigenetically stable (no relevant escape mutants) [30–32]. Antisera from individuals infected decades ago retain the ability to neutralise current wild-type (WT) strains of MeV and vice versa, although with different efficiency. In the recent measles outbreaks, MeV strains of non-A genotypes, in particular type D, have been isolated [33–35]. Nevertheless, they can still be neutralised by anti-H antisera without significant restriction, indicating that the vaccines are still fully effective [30, 31]. The vast majority of measles patients in current outbreaks had not or not been sufficiently vaccinated, especially in terms of the second dose [10, 35, 36].

(2) Are the current vaccination programmes appropriate?

The timing of delivery strategies for the first and second vaccination dose varies across countries and regions. In countries with ongoing transmission, WHO recommends the administration of the first dose at the age of nine months to ensure optimal protection during the susceptible period in infancy. Re-vaccination is recommended in the second year of life (15–18 months) [11] to booster and prolong a strong immunity as well as to immunise individuals in which the first vaccination has failed (primary vaccine failure, see above). The minimum interval between the first and second dose is one month. Catch-up vaccination is recommended for all children, adolescents and adults, who have not received the first or second dose of MMR vaccine or have lost their vaccination records in most countries. However, in many European countries those catch-up vaccination programmes were not conducted or accepted efficiently. As a result, measles outbreaks are still occurring despite significantly increasing vaccination rates with a majority of adolescents and young adults (up to 40 years of age) being affected, who were mainly not vaccinated against measles or had received only one dose. In addition, a high percentage of this age group is also not naturally immune against measles [37]. This lack of immunity leads to another complex problem: pregnant women of childbearing age, who were not protected against measles themselves, cannot provide vertical protection for their infants [37]. Furthermore, in the current epidemiological situation they are at risk to acquire measles in pregnancy, which is associated with a higher incidence of hospitalisation, measles-related complications (e.g. pneumonitis) including maternal death and adverse pregnancy outcomes like pregnancy loss, preterm birth and low birth weight, but not congenital defects [38–40]. Infections shortly before or after delivery can lead to intrauterine, perinatal or postnatal MeV infections of the newborn, who then have a high risk to develop SSPE due to their immature immune system [5, 8, 41, 42]. Because of all these aspects mentioned above, it has to be discussed, if additional efforts and other vaccination schedules are necessary to close the vaccination gaps [37].

Other setbacks why successful vaccination programmes were interrupted leading again to large vaccination gaps are catastrophes including life-threatening disease outbreaks like the Ebola epidemic in West Africa and wars (e.g. in Syria). Currently millions of refugees live in crowded camps or are travelling thousands of kilometres through different countries (part of them with ongoing large measles outbreaks, e.g., in Bosnia-Herzegovina, Serbia) to reach Central and Northern Europe. Because of the mass accumulations of migrants and the rapid migration movements, there is a high risk of transmission and wide spread of infectious diseases, especially measles [43]. There is no possibility to vaccinate these masses of refugees during their migration on the transit routes; however, in the accommodating countries like Germany and Austria, the MMR vaccination of refugees has the highest priority, although the organisation of those mass vaccinations is still a big challenge. In parallel, a rapid increase in the MMR vaccination rates of the native population is necessary to reduce the risk of large outbreaks.

How many people must be vaccinated to establish stable herd immunity for the population preventing virus transmission and mediating protection for individuals who are not immune? This depends on the basic reproduction rate (R_0_), which indicates how many people on average are infected by an initial spreader in a fully susceptible population. For measles, the index has been calculated to be 12–18, which is one of the highest for any human pathogen [24]. This demands that >94 % of a population has to be immune to stop virus spread (and to ensure herd immunity) [5, 9]. This high rate has been reached for smallpox, when the vaccination had been regulated by law in the most countries. Without such regulation, wild poliovirus type 2 (PV-2) has been eradicated. The eradication of PV-3 is nearly reached [44]. However, PV-2 vaccine-derived strains and wild poliovirus type 1 strains are still circulating in some countries (Israel, West Africa, Afghanistan and Pakistan) [45–47]. The basic reproduction rate (R_0_) of poliovirus was calculated 4–13 [9]. Consequently, an immunity rate of about 85 % was necessary to effectively interrupt virus circulation. This was reached by vaccination against poliovirus type 2, while the rates for poliovirus 1 and 3 have been found to be sometimes lower [48]. However, a worldwide system of surveillance discovers and closes the gaps in all countries in which vaccination is strictly applied. To exceed a vaccination rate of 90 %, without regulation by law, is a big challenge for the public health system and seems like Sisyphean labour. Measles outbreaks happened when this line was underrun. For these reasons, additional efforts are absolutely necessary to increase vaccine acceptance.

Potentially alarming are, however, reports on sporadic cases of overt measles disease amongst adults, who received two doses of measles vaccine decades ago [5, 38, 49–52]. Some of the cases led to further transmission events and occurred typically in countries which have implemented their vaccination programmes early and successfully and thus achieved the WHO elimination goals, i.e. complete elimination of MeV transmission. These cases could indicate that vaccine-induced IgG levels are waning [20, 53, 54] and the immunological memory to MeV is more limited in the absence of wild-type MeV exposure events than thought before. If cases of breakthrough measles due to secondary vaccine failure are seen more often, this should be an incentive to enhance the speed of the elimination process!

(3) How to speed up the elimination process and arouse public opinion? Should vaccination be regulated by law?

After smallpox, no other vaccination has been so far regulated by law globally, since no other ubiquitously spread infectious disease was considered so life-threatening, especially when an anti-infective therapy is missing. The vaccination against smallpox produced many and severe side effects. The more smallpox waned, the more the vaccination was criticised and refused. Although the anti-MeV vaccination produces much less side effects, it becomes more and more neglected or even rejected when—as a consequence of successful vaccination programmes—the dangerous measles complications decline and disappear out of the public attention, while the fear of adverse effects is increasing [55]. Other factors that have an impact on vaccine uptake in countries with high socio-economical standards are complacency, lack of education about the seriousness of the disease and a degree of mistrust in the medical establishment and the pharmaceutical industry [1, 9]. This results in an increasing number of parents, who are sceptical towards vaccination [9, 56, 57]. In opposite to oral vaccines, injections are being perceived as unpleasant for children and parents. Therefore, combining several vaccines in one injection has proven to be a convenient approach. Such a combined vaccine has been introduced for measles, rubella and mumps and has been extended to varicella (MMR, respectively, MMRV vaccine). It has revealed that the immunogenic infectivity of the single attenuated viruses is not impaired [58]. The combined vaccine should be applied not only for the primary immunisation, but also for secondary boosters to close immunity gaps deriving from the first vaccination. It has been proven very effective in elimination of poliomyelitis caused by three immunologically different poliovirus types [59]. Complications of poliovirus infections are no more frequent than those from measles, but patients suffering from side effects such as paralysis are living amongst us as a visible and constant reminder of this issue. In opposite to poliomyelitis, measles are frequently considered as an unpleasant, but healing up disease of children, even by some medical doctors who believe that passed measles strengthens the power of resistance. However, measles impair the immune system for a longer period of time in which other infectious diseases may happen. So, the public attention on measles must be enhanced and kept high aside of current measles outbreaks [9, 10].

(4) Is the measles surveillance system adequate?

Smallpox has been eradicated not only by vaccination, but also by an effective global reporting and surveillance system. Also for measles (and rubella) elimination a rapid, exact and sensitive surveillance system is of major importance. For the national health authorities, it is important that clinically suspected cases were reported rapidly and implemented control measures can be executed immediately. However, the virological surveillance and laboratory performance play an essential role. In countries near elimination, all suspected cases should be routine laboratory confirmed and PCR samples of anti-MeV-IgM-positive samples sent to a national reference laboratory for further investigation. In some cases, interpretation of laboratory results is challenging (e.g. in persons with a recent history of vaccination, false-positive results because of cross-reactivity with other infections, indeterminate test results or positive results for both measles and another virus like Parvovirus B19, Epstein Barr virus or human herpesvirus type 6). For the elucidation of transmission chains, the differentiation of endemic circulation or the importation of MeV strains, differentiation of imported/imported-related measles cases and the interruption of transmission highly sensitive virological and molecular biological test methods (i.e. viral sequencing, genotyping and phylogenetic analyses) are essential tools for the assessment of epidemiological situation [60, 61].

Measles patients are on average most infectious four days before and after the onset of exanthema. Measles are a severe respiratory disease producing epidemiological issues similar to influenza. Global tourism and worldwide migration due to war and poor economic conditions pose additional challenges to the WHO project of measles eradication [9, 62]. Currently, Europe is affected by massive flows of refugees enhancing, in combination with an increasing sceptical attitude of the Central European population towards vaccines, the risk of new outbreaks. Apart from that, in industrial countries, where measles had become rare, doctors are not so familiar with the clinical symptoms. In underdeveloped countries, malnutrition makes measles more severe and prolongs the period of infectivity. It has to be discussed whether immigrants and tropical tourists should be checked on measles immunity by antibody tests. Simple methods focus on oral fluid swabs saving blood sampling. However, with the current large migration streams in Europe, this would be not feasible, but the immediate administration of the MMR vaccine when the refugees reach their target country has a high priority. Reporting obligation has to be established and expanded in the public health system of every country. Modern molecular diagnostic tools are available for rapid tracing infectious chains [33, 63].

## Conclusions

Measles can be best prevented by vaccination. Live-attenuated MeV strains, preferentially strain Enders/Schwarz, are used. Although they had been developed more than 50 years ago, the currently used vaccines are very effective for establishing immune protection which lasts for several decades when the infants were vaccinated twice in their second year of life. MeV breakthrough infections have been recorded as exception of this rule. A large majority of measles outbreaks in Europe which happened in the last five years have been caused by immunity gaps resulting from deficient participation in the vaccination programmes. More than 95 % of a population must be successfully vaccinated to stop MeV circulation and to prevent outbreaks after MeV re-import from countries in which measles are still endemic. These countries suffer from socio-economic problems, political riots, etc. and need special support from WHO and charity organisations. Paradoxically, also in developed countries the necessary rate of vaccine-immunised people is underrun in some regions. This is reasoned by insufficient information on life-threatening complications due to measles. As a consequence of successful vaccination programmes, the public attention to this problem decreases even in medical doctors, who are today less familiar with this disease. Measles vaccine combined with rubella, mumps and possibly varicella will probably meet a bigger acceptance in the population. More sentinel programmes of laboratory immunity checks should be established to discover gaps in herd immunity as early as possible [36]. If we succeed in increasing the acceptance of the measles vaccination and the awareness of the risks of MeV infections in our population, we really could—with the assistance of the effective combined vaccines—approach the major goal of regional measles elimination/worldwide eradication. Vaccination and surveillance programmes had successfully led to the eradication of poxviruses and had proven very useful in fighting the spread of poliomyelitis. Morbillivirus vaccines are effective, as the eradication of rinderpest virus (2010) has shown [64].
